# Virulence factors in coagulase-positive staphylococci of veterinary interest other than *Staphylococcus aureus*

**DOI:** 10.1080/01652176.2020.1748253

**Published:** 2020-04-17

**Authors:** Margarita González-Martín, Juan Alberto Corbera, Alejandro Suárez-Bonnet, María Teresa Tejedor-Junco

**Affiliations:** a Research Institute of Biomedical and Health Sciences, University of Las Palmas de Gran Canaria, Las Palmas de Gran Canaria, Canary Islands, Spain; b Department of Pathobiology and Population Sciences, The Royal Veterinary College, Hatfield, United Kingdom

**Keywords:** veterinary, zoonosis, coagulase-positive Staphylococci, non-aureus Staphylococci, virulence factors, toxins, biofilm, surface proteins

## Abstract

Coagulase-positive Staphylococci (CoPS) can exist as commensals in humans, companion and food-producing animals, but can cause severe or even lethal diseases. Exchange of these bacteria between humans and animals has been described. Special attention has been focused on Methicillin-Resistant *Staphylococcus aureus*, but other CoPS can also represent an important threat. In addition to significant antimicrobial resistance, these bacteria may carry a plethora of virulence factors - molecules that allow bacteria to establish on or within a host and increase their ability to cause disease. These virulence factors have been widely described in *S. aureus* but information about other species of CoPS is scarce. The aim of this paper is to review the recent literature about the virulence factors of non-aureus CoPS of animal origin. Their possible effects on human health are also described. The role and prevalence of different virulence factors including leukocidins, hemolysins, adhesins, enterotoxins, exfoliative and toxic shock syndrome toxins as well as superantigen-like proteins are addressed. The effect of these virulence factors on human health is also described. The possibility of misdiagnosis of species of CoPS has been demonstrated in human clinical samples. Prevalence of zoonotic infections could be higher than thought and medical laboratories should be aware of these other staphylococcal species. In keeping with the ‘One Health’ approach to animal and human disease, medical professionals, veterinarians and health workers should be aware of the risks derived from exposure to these bacteria in people in close contact with animals, including pet owners, farmers and veterinarians themselves.

## 1. Introduction

*Staphylococcus* is a bacterial genus composed of non-motile facultative anaerobic Gram positive cocci that appear as clusters under microscopic examination and are, with some exceptions, catalase positive. Staphylococci are usually differentiated into two groups using the coagulase test. It is assumed that coagulase positive staphylococci (CoPS) are usually pathogenic, even when in some cases they can cause asymptomatic colonization in healthy individuals, and coagulase negative (CoNS) are saprophytic or cause opportunistic infections. In veterinary bacteriology, nine species of CoPS are generally recognized: *Staphylococcus aureus* (including subsp. *anaerobius*) (De La Fuente et al. 1985), *Staphylococcus intermedius* (Hajek 1976), *Staphylococcus pseudintermedius* (Devriese et al. 2005), *Staphylococcus delphini* (Varaldo et al. 1988), *Staphylococcus hyicus* (Devriese et al. 1978), *Staphylococcus schleiferi* subsp. c*oagulans* (Igimi et al. 1990), *Staphylococcus lutrae* (Foster et al. 1997)*, Staphylococcus agnetis* (Taponen et al. 2012) and *Staphylococcus cornubiensis* (Murray et al. 2018). Some species, like *S. hyicus* and *S. agnetis* are defined as coagulase variable (meaning that some strains can be coagulase positive and some coagulase negative).

The application of molecular typing tools has changed the taxonomy of the *Staphylococcus* genus allowing the differentiation of species previously indistinguishable using phenotypic tests (Sasaki et al. 2007; Blaiotta et al. 2010; Sasaki et al. 2010; Decristophoris et al. 2011) and leading to a reclassification of most *S. intermedius* canine isolates that were renamed as *S. pseudintermedius* (Ross Fitzgerald 2009; Bond and Loeffler 2012). *S. intermedius*, *S. delphini* and *S. cornubiensis* constitute the so-called *Staphylococcus intermedius* group (SIG) (Sasaki et al. 2007; Bannoehr et al. 2007; Murray et al. 2018). For routine diagnostic microbiology purposes, phenotypic tests, combined with information about the host (due to high host specificity) are likely to be sufficient, but for research applications, precise identification to species level is needed.

Our review focuses on virulence factors in coagulase positive Staphylococci isolated from animals. *S. aureus* is not discussed in this paper due to the existence of a number of recent review articles covering this species; see for example (Zecconi and Scali 2013; Scali et al. 2015; Mutters et al. 2016; Ballhausen et al. 2017; Aires-de-Sousa 2017; Rainard et al. 2018).

Knowledge about virulence factors and the pathways that regulate their production could help to design new therapeutic or vaccination approaches (Scali et al. 2015; Kong et al. 2016).

## 2. Main virulence factors described in different species of Staphylococci

Staphylococci can have a wide variety of virulence factors that allow the bacteria to avoid the immune system and contribute to increased severity of infections. Most of these factors have been initially described in *S. aureus* and include surface proteins (Protein A, clumping factor, fibronectin binding proteins or iron regulated surface determinants), capsular polysaccharides, molecules involved in biofilm formation (for example polysaccharide intercellular adhesin) or toxins (pore-forming toxins, toxins that act as superantigens). Cell wall adhesins that recognize extracellular matrix proteins are also called MSCRAMMS (microbial surface components recognizing adhesive matrix molecules). Some enzymes (coagulase, staphylokinase and proteases) also contribute to immune evasion and host tissue penetration (Zecconi and Scali 2013; Scali et al. 2015; Ballhausen et al. 2017; Seilie and Bubeck Wardenburg 2017).

Surface proteins are involved in adhesion, tissue invasion and evasion of immune defense. Among the virulence factors that contribute to evade immune response, staphylococcal protein A has a key role. Almost all *S. aureus* isolates synthetize this protein, which binds immunoglobulins, inhibits opsonization and phagocytosis, and acts as a superantigen (Balachandran et al. 2018). Clumping factors A and B are also important for adhesion and immune defenses evasion. They bind to fibrinogen facilitating host tissues invasion. Fibronectin binding proteins are able to bind fibronectin and elastin present in the extracellular matrix, promoting invasion of host tissues. Iron regulated surface determinants are involved in staphylococcal iron uptake systems (Scali et al. 2015).

Biofilms are bacterial populations enclosed in an organic matrix. They promote adhesion to surfaces and provide protection against antibiotic and immune defenses (Zecconi and Scali 2013).

Pore forming toxins (PFTs) have been widely studied in *S. aureus*. Their main activity is to disrupt eukaryotic membranes, causing cell lysis. Based on the transmembrane components structure, these toxins have been divided into two groups: α-PFTs and β-PFTs. In *S. aureus,* seven *β-*PFTs have been described (one α-hemolysin and six bicomponent leukocidins) (Seilie and Bubeck Wardenburg 2017). They have different host cell targets (monocytes, neutrophils, platelets, erythrocytes and epithelial cells). Panton-Valentine leukocidin (PVL) has been reported to be almost 100 times more potent than the other staphylococcal leukocidins (Kong et al. 2016).

Among the toxins that act as superantigens, we include pyrogenic toxins (staphylococcal enterotoxins (SEs) and toxic shock syndrome toxin (TSST)) (Kong et al. 2016; Ballhausen et al. 2017). More than 20 SEs have been identified to date, and are one of the most frequent causes of food-borne disease. TSST-1 causes a serious illness with high mortality. Both, SEs and TSST-1, act as superantigens triggering T-cell activation and proliferation, and activating cytokine release and cell death.

Exfoliative toxins (ETs) cause staphylococcal scalded skin syndrome characterized by destruction of desmosomal cell attachments (desmoglein-1) resulting in detachment of the epidermis.

In other *Staphylococcus* species, information about virulence factors and their regulation is much sparser.

## 3. Virulence factors described in different species of coagulase positive staphylococci

### 3.1. Staphylococcus intermedius group

As mentioned before, four species are included: *S. intermedius*, *S. pseudintermedius, S. delphini* and *S. cornubiensis*. Sasaki et al. (2007) and Bannoehr et al. (2007) analyzed collections of strains of “*S. intermedius*” from Japan and Europe. They found that all the isolates from human beings, dogs and cats were *S. pseudintermedius* meanwhile most isolates from feral pigeons were *S. intermedius* and most strains isolated from horses and domestic pigeons were *S. delphini.* Some authors describe virulence factors in canine isolates of *S. intermedius* that, following the revised taxonomy, can be considered as belonging to *S. pseudintermedius* (Futagawa-Saito et al. 2006; Schmidt et al. 2009). Futagawa-Saito et al. (2006) describe isolates from pigeons that were clearly different from canine isolates, so due to host specificity we could assume they were truly *S. intermedius* or *S. delphini.* On the other hand, a recent publication (Velázquez-Guadarrama et al. 2017) describes results that clearly contradict this hypothesis, reporting 86% *S. intermedius* and 14% *S. pseudintermedius* CoPS isolates among dogs. Strains were identified using 16S rRNA sequencing, but these proportions could be biased because they only tested mannitol-positive isolates and, in *S. pseudintermedius* acid production from mannitol is weak and delayed (Devriese et al. 2005). This fact does not affect the description of virulence factors because identification was appropriate.

Several authors have described virulence factors related to adhesion and tissue invasion in isolates of SIG. In a study, using *S. pseudintermedius* isolated from healthy dogs and dogs suffering atopic dermatitis, no statistical differences in adherence between isolates from atopic or healthy skin were found. This suggests that host factors, rather than bacterial virulence, are the cause of increased bacterial adherence to the atopic canine skin (Schmidt et al. 2009). Bannoehr et al. (2012) characterized two surface proteins of *S. pseudintermedius* (SpsD and SpsO) that mediate adherence to canine keratinocytes contributing to skin colonization and infection. They found that the SpsO mediated adherence took place with keratinocytes of all dogs used in the study but SpsD mediated adherence was more variable, suggesting variability of presence of receptors for Sps-D in different animals. Invasion of canine keratinocytes is promoted by two fibronectin binding proteins, SpsD and SpsL, anchored in the bacterial wall (Pietrocola et al. 2015).

Bannoehr et al. (2011) showed that the protein SpsQ found in *S. pseudintermedius* was analogous to protein A in *S. aureus*. In addition, they found that the protein SpsP had 73% homology to SpsQ at amino acid level. Balachandran et al. (2018) observed that isolates of *S. pseudintermedius* harbor and express genes analogous to those that code for protein A in *S. aureus* and, demonstrated that canine IgG recognizes and binds to protein A on *S. pseudintermedius*, predominantly via its Fc region. The activity of this protein as a superantigen has not been studied and its toxic effect on B-cells are yet to be determined.

Futagawa-Saito et al. (2006) studied *S. intermedius* isolates obtained from pigeons and from dogs. Clumping factor and Protein A were not detected in any *S. intermedius* isolated from pigeons. Protein A and clumping factor were detected in 54.5% of canine isolates, almost all considered now *S. pseudintermedius*.

In 15 isolates of *S. intermedius,* obtained from cases of caprine subclinical mastitis, the most frequent genes encoding surface proteins involved in adhesion were laminin, elastin and fibrinogen binding proteins, in addition to one for fibronectin binding protein A (Salaberry et al. 2015). Genes encoding collagen binding protein and fibronectin binding protein B were not detected, and the *bap* gene, encoding biofilm-associated proteins was observed in only 13% of isolates. However, it is necessary to point out the lack of precision of the method used in this work, for the identification of species belonging to SIG, so these results should be assumed with caution.

Biofilm formation has been described as significantly higher in *S. pseudintermedius* than in *S. intermedius* isolates (Futagawa-Saito et al. 2006; Velázquez-Guadarrama et al. 2017). Few studies on genes and mechanisms involved in biofilm production have been found. Casagrande et al. (Casagrande Proietti et al. 2015) studied biofilm-forming ability in 60 *S. pseudintermedius* isolates from dogs with pyoderma. Genes *ica*A and *ica*D, that code a polysaccharide intercellular adhesion molecule (PIA) were detected in 91% of isolates and all isolates contained either both genes or had neither of them. Phenotypic tests used were Congo Red Agar test (CRA), tube adherence test and Microtiter plate test (MtP) with the results demonstrating good agreement between methods. Three of the strains harboring the genes were non-biofilm producers according to the three phenotypic methods. Four strains were found to be biofilm producers by MtP and tube adherence test but they did not possess *ica*A and *ica*D genes, suggesting that they could form biofilms without polysaccharide intercellular adhesion molecules. In a previous study (Singh et al. 2013), a similar proportion of biofilm producer strains was observed but some isolates with only *ica*A or *ica*D genes were detected and more variability between phenotypic and genotypic methods was obtained.

Comparing the presence of some virulence factors on *S. pseudintermedius* obtained from healthy and diseased dogs could help to understand their pathogenic role. Garbacz et al. (2013) compared their presence in 71 isolates from healthy dogs and 120 from diseased dogs and found that production of Protein A was more frequent in infected dogs. This was the only factor that showed a statistically significant difference. No differences between both groups were found for biofilm formation, coagulase, clumping factor, DNase and lipase.

Comparisons have also been made between Methicillin-susceptible (MSSP) and Methicillin-resistant *S. pseudintermedius* (MRSP) in order to determine if their virulence factor profiles are different. In another study (Couto et al. 2016), twenty one MRSP and twenty one MSSP, obtained from 18 asymptomatic carriers and 24 diseased dogs and cats were included. MRSP seemed to upregulate surface proteins, increasing the adherence of isolates to keratinocytes. MRSP and MSSP have the capacity to form biofilms, but MSSP showed an increased ability to produce biofilm under acidic conditions. No differences in biofilm formation were found between clinical isolates and isolates from carriers.

Using multi-locus sequence typing (MLST) for *S. pseudintermedius,* Bergot et al. (2018) determined that the sequence types (ST) 71, ST258 and ST496 are the most prevalent in France. Each of them possesses a specific pattern of virulence genes. All MLST types shared a core virulence profile that includes the biofilm-associated *ica* operon and *sps* surface proteins. The cell wall associated genes *spsI* were detected only in isolates belonging to ST496.

Diribe et al. (2015) compared virulence gene profiles between multidrug resistant (MDRSP) and multidrug susceptible *S. pseudintermedius* associated with postoperative surgical canine infections. Biofilm production was assayed using microtiter plate technique and all isolates formed biofilms in different quantities, but no significant difference between MDRSP and non-MDRSP were found.

Arginine catabolic mobile element (ACME) is a mobile element described in *S. aureus* that contains the *arc* gene. This gene encodes an arginine deaminase system that facilitates colonization of human skin. Until the study of Yang et al. (2017), ACME had not been reported in *S. pseudintermedius*. Among Methicillin-resistant and Methicillin-susceptible *S. pseudintermedius* isolates from dogs and cats, a similar prevalence (70%) of carriage of ACME-*arcA* was found. ACME-*arcA* positive isolates were obtained more frequently from skin samples than from other body sites, suggesting that it could provide an advantage for colonization of animal skin.

Staphylococcal Pore-forming toxins include hemolysins and leukocidins. Futagawa-Saito et al. (2006) reported that all S. *intermedius* isolates from pigeons were hemolytic on sheep red blood cells (RBCs), but only 17.7% showed hemolytic activity on rabbit RBCs or possessed protease activity. Almost all their canine isolates, considered now *S. pseudintermedius*, caused hemolysis of sheep and rabbit RBCs. No differences on β-hemolysin production between healthy and diseased dogs were noticed (Garbacz et al. 2013).

The pathogenic role of several virulence factors of *S. pseudintermedius* was studied by Garbacz et al. (2013) comparing their presence in 71 isolates from healthy dogs and 120 from diseased dogs. Surprisingly, all the strains included in Garbacz et al. (2013) study, from healthy and infected dogs, possessed leukotoxin genes *lukS*/F. Virulence factor profiles of 196 MRSP isolated from healthy dogs (Gómez-Sanz et al. 2011) showed that all of them, independent of their genetic background, carried *lukS/F-I*. In a later study (Gómez-Sanz et al. 2013), it was shown that all isolates of MSSP (n = 16) also carried *lukS/F-I*. The *Luk-I* gene was detected in 29% isolates of *S. pseudintermedius* obtained from diseased dogs in Lithuania (Ruzauskas et al. 2016) and in more than 90% isolates of clinical samples from domestic animals in Brazil (Pitchenin et al. 2018).

PCRs were performed to detect *lukS, lukF* and Panton-Valentine leukocidin genes, comparing multidrug resistant and multidrug susceptible *S. pseudintermedius* associated with postoperative surgical canine infections (Diribe et al. 2015). No differences in carriage of these virulence factor genes, between both groups of *S. pseudintermedius,* were observed.

Abouelkhair et al. (2018) first characterized a leukocidin (Luk-I), very similar to PVL, from a staphylococcal species other than *S. aureus*, which had its main effects against canine neutrophils. Antibodies produced in dogs against attenuated Luk-I reduced the cytotoxic activity and could constitute an important tool in the prevention and control of *S. pseudintermedius* infections (Abouelkhair et al. 2018). Maali et al. (2018) proposed that *S. pseudintermedius* virulence could be explained by the selective action of Luk-I on immune cells expressing specific receptors, combined with the action of pro-inflammatory and cytolytic staphylococcal peptide toxins (PSMs).

Garbacz et al. (2013) found exfoliative toxin (ET) genes in all the strains of *S. pseudintermedius* included in their study, obtained from healthy (71 isolates) and diseased dogs (120 isolates). Previously, Futagawa-Saito et al. (2009) detected a novel exfoliative toxin gene (*exi)* coding the first exfoliative toxin in *S. pseudintermedius* (EXI). Its sequence shares a significant homology with the ones of other staphylococcal ET, especially with ETB from *S. aureus*. It was found to have a prevalence of 23.3% in isolates from canine pyoderma. Crude supernatant proteins from *S. pseudintermedius* harboring this gene caused characteristic skin exfoliation in neonatal mice 6-8 hours after injection. Iyori et al. (2011) produced a recombinant EXI by *E. coli* expression using an *exi* gene obtained from *S. pseudintermedius* isolated from a dog suffering impetigo. When injected intradermally in healthy dogs, recombinant EXI caused vesicles or erosion with superficial epidermal splitting. By immunofluorescence analysis, activity of EXI on desmoglein-1 of canine skin was demonstrated. In the Garbacz et al. (2013) study, the exfoliative toxin gene tested was *siet* and not *exi.* SIET is a toxin, encoded by the *siet* gene, obtained from chromosomal DNA of *S. intermedius*. Iyori et al. (2011) found that SIET did not induce epidermal splitting. From the results of both studies, it can be suggested that EXI, but not SIET, participates in canine epidermal splitting. SIET probably has a role in canine pyoderma and chronic otitis (Yoon et al. 2010; Youn et al. 2011; Bardiau et al. 2013; Ruzauskas et al. 2016). Almost all the isolates from otitis and dermatitis in domestic animals included in a recent study (Pitchenin et al. 2018) produced SIET toxin and 24% of isolates produced EXI exfoliative toxin. The possibility that SIET-producing *S. pseudintermedius* can cause ophthalmic disease has also been suggested (Kang et al. 2014). In a study examining further exfoliative toxin genes in *S. pseudintermedius* (Iyori et al. 2010), a novel open-reading frame (ORF) was found in the genome of dogs with impetigo. The deduced amino acid sequence had 70.4% homology with that of SHTEB exfoliative toxin from *S. hyicus*.

Tabatabaei et al. (2019) found that of 19 MRSP isolated from dogs (12) and cats (7), all of them possessed *siet* genes, 15 harbored *exp*A, 6 *sea* and only one *exp*B. Other studies found also that almost all isolates harbor *siet* genes (Gómez-Sanz et al. 2011; Gómez-Sanz et al. 2013). In the later work, 15 isolates harbored exfoliative toxin gene *exp*A and only one of them, *exp*B.

Knowledge about *S. pseudintermedius* enterotoxins (SEs) and their possible relation to digestive pathologies is scarce. Many isolates of SIG group harbor SEs genes (Gómez-Sanz et al. 2011; Gómez-Sanz et al. 2013).

The role of *S. pseudintermedius* enterotoxins (SEs) in the pathogenesis of pyoderma has been investigated (Tanabe et al. 2013) and no differences between healthy and diseased dogs were detected, suggesting that these toxins do not participate in the pathogenesis of this disease. The gene *sec* was the only one found in *S. pseudintermedius* strains isolated from carriers and infected dogs and at a very low prevalence (1.6%) (Garbacz et al. 2013). When different *S. pseudintermedius* isolates from different clinical samples (mainly canine) were studied, a much higher prevalence of the enterotoxin genes *sec* (57%) and *sea* (17%) was observed (Pitchenin et al. 2018). Considering otitis and dermatitis isolates (39), 24 harbored the *sec* and 6 the *sea* genes. The gene *sec_canine_* (canine type C staphylococcal enterotoxin) is more frequent in isolates from dogs with cutaneous infections (Yoon et al. 2010), usually clustered with *sel*.

In 15 *S. intermedius* isolated from subclinical mastitis, all genes encoding enterotoxins (A-E) were detected, with *sec* the most frequent (n = 7) but only 6% (n = 1) each of the others (Salaberry et al. 2015). Due to their potential to cause vomiting and diarrhea, the presence of enterotoxins in non-*aureus* staphylococci could also be considered a risk in food-producing animals. As mentioned before, the method used for identification may give incorrect results and, in addition to this, most of the genes were found in one unique isolate.

In healthy dogs, high prevalence of nasal carriage of virulent *S. pseudintermedius* has been observed (Gharsa et al. 2013). Fifty-five isolates were tested for 35 virulence genes, including genes encoding for staphylococcal enterotoxins, leukocidins, hemolysins, exfoliative toxins (*siet, exp*A, *eta, etb* and *etd)* and the *tst* gene encoding for the toxic shock syndrome toxin. A large number and variety of virulence factors were detected in this study and no relation with genetic background or resistance patterns was observed. This result is in line with others (Gómez-Sanz et al. 2013) that reported the presence of these virulence factors in healthy dogs and their owners.

Studies on virulence factors of *S. delphini* are scarce. Sudagidan and Aydin (2012) studied 18 isolates of *S. delphini* obtained from domestic pigeons; 50% of them were biofilm producers but none were found to harbor the virulence genes analyzed (enterotoxins, TSST, leukocidins, PVL, hemolysins, exfoliative toxins). *S. aureus*-specific primers were used, so lack of sufficient sequence similarity could be an explanation for these results. In Tunisia, 19% of healthy donkeys were reported to carry *S. delphini* (Gharsa et al. 2015) and *lukS-I* and *siet* genes were detected in all 19 isolates. The gene *se-int* was detected in 17 isolates, *sec_canine_* in four isolates and *exp*A in one. *LukF* was not observed in any of the isolates. In 12 isolates from wild birds, Ruiz-Ripa et al. (2019) found similar results; all of them presented genes *lukS-I, siet* and *se-int* but two of them also contained the leukocidin gene, *lukF-I.*

Due to the low number of isolates included in all these studies, it is not possible to determine if the absence of some virulence factors like *lukF-I* is a characteristic of this species or could vary depending on geographical region or animal species.

### 3.2. Staphylococcus hyicus

*S. hyicus* is the causative agent of exudative epidermitis in pigs, a disease with a worldwide distribution and it has been reported to cause mastitis in cattle. Two groups of *S. hyicus* can be considered based on the ability to cause disease. Isolates obtained from diseased pigs produce different exfoliative toxins (Exh) (Andresen et al. 1997), that have been designated as SHETA and SHETB (Sato et al. 2000) and ExhA, B, C and D (Ahrens and Andresen 2004). Exfoliative toxins of *S. hyicus* are serine proteases targeting desmoglein-1 protein, thereby causing destruction of desmosomal cell attachments and detachment of the epidermis (Nishifuji et al. 2008). SHETB also contains a serine-protease like sequence (Watanabe et al. 2000), absent in SHETA, but their effect on desmoglein-1 has not been tested (Nishifuji et al. 2008).

The prevalence of genes encoding exfoliative toxins in *S. hyicus* isolates from various countries have been studied (Sato et al. 2000; Ahrens and Andresen 2004; Futagawa-Saito et al. 2007; Kanbar et al. 2008) and it seems that the carriage of these genes varies depending on the geographic area. The carriage of exfoliative toxins genes appears to be significantly higher in isolates from diseased pigs (86.6%) than in those from healthy animals (19.6%) (Futagawa-Saito et al. 2007).

SHETA is encoded chromosomally but SEHTB is encoded on a plasmid (Sato et al. 2000). A genomic island harboring toxin-encoding genes has been described in *S. hyicus* ATCC11249 strain (Calcutt et al. 2015). In addition to a cluster of five genes encoding ExhA toxin, an ORF with a 64% identity to the epidermal cell differentiation inhibitor toxin (EDIN) of *S. aureus* was found. A delta hemolysin, a type VII secretion system and a putative gas vesicle protein gene cluster were found in other genomic locations. When a genome comparison among toxigenic and non-toxigenic *S. hyicus* strains was made (Leekitcharoenphon et al. 2016), two genomic regions that are predicted to encode virulence factors related with exudative epidermitis (including *Exh*A and EDIN genes) were predominantly observed in toxigenic strains.

A protein with similar characteristics to *S. aureus* protein A has been described in *S. hyicus* type strain CCUG 15602/ATCC 11249 (Rosander et al. 2011). This protein had four IgG binding domains (instead of the five described in *S. aureus* protein A). In clinical isolates of *S. hyicus* the *spa* gene had either three or four domains, independent of their origin.

### 3.3. Staphylococcus agnetis

After the isolation of the new species *S. agnetis* from mastitic milk in Finland (Taponen et al. 2012), genotypic analyses indicated a close relation with *S. hyicus*, raising the possibility that some *S. hyicus* isolates may have been *S. agnetis*. Calcutt et al. (2014) determined a draft genome sequence of an *S. agnetis* isolate from mastitic milk in Canada. Some ORFs related to potential virulence factors were found, two with a significant similarity to staphylocoagulase and one with a significant similarity to beta toxin of *S. aureus*. In addition to this, three clustered genes encoding putative superantigens and one gene encoding a putative hyaluronidase were detected, the latter significant, because among staphylococci, hyaluronidase genes have only been described previously in *S. aureus*. Two isolates of coagulase-negative *S. agnetis* obtained from milk samples of mastitic cows were studied to determine the presence of several virulence genes. The exfoliative toxin gene *etd* was observed in one isolate and γ-hemolysin in two (Mahato et al. 2017). The presence of genes encoding for enterotoxins *seb* and *sec,* and for toxic shock syndrome toxin *tst1*, in an isolate of *S. agnetis* obtained from milk has also been described (Rahmdel et al. 2018).

Four *S. agnetis* isolates were included in an extensive study of putative virulence factors of staphylococci causing bovine mastitis (Åvall-Jääskeläinen et al. 2018). Whole genomes were obtained and compared with the Virulence Factor Database. All the isolates contained beta-toxin, hemolysin III and 3 out of 4 exfoliative toxin A, with none containing leukotoxins. Among superantigen and superantigen-like protein genes, only *set*7 and one unnumbered exotoxin gene were detected, both in all the isolates. Multiple MSCRAMM genes were detected in all isolates: collagen adhesin; elastin, fibrinogen and fibronectin-binding proteins; enolase; protein A; surface proteins sasF and sasH; and biofilm associated protein genes. Exoenzyme encoding genes detected in all four genomes included the ones for catalase, staphylocoagulase, hyaluronidase, Glyceraldehyde 3-phosphate dehydrogenase, von Willebrand factor binding protein, aureolysin and cysteine proteinase A.

In 2015, *S. agnetis* was reported as causative agent of lameness in chickens (Al-Rubaye et al. 2015). The whole genome was sequenced and compared with the one of other pathogenic Staphylococci, which revealed that *S. agnetis* contains a distinct repertoire of virulence determinants. In agreement with Calcutt et al. (2014) a hyaluronidase gene was detected but five (instead of three) superantigen like proteins. An exfoliative toxin A orthologue was found that had not been previously described in the draft genome of the isolate from mastitis. The *S. agnetis* genome also encodes seven fibronectin-binding proteins. To determine which genes might mediate infection in chickens, predicted *S. agnetis* proteins were compared to the Virulence Factor Database. Several virulence factors (17 for host immune evasion, 11 for host adherence, 7 for toxins biosynthesis and 5 secretion systems) were identified.

*S. agnetis* was isolated in pure culture from 16 of 997 broiler breeders found dead (Poulsen et al. 2017), with lesions of endocarditis and septicemia. In the same flocks, the bacterium was also isolated in pure culture from the cloacal microbiota of newly hatched chickens. The whole genome sequence from three isolates was obtained to identify possible virulence genes. Seven fibronectin binding protein-encoding genes and five enterotoxin-associated genes were identified.

### 3.4. Other coagulase positive staphylococci

Information about virulence factors in other species of CoPS is scarce. Osman et al. (2016) described virulence factors detected in four isolates of *S. schleiferi* subsp. *coagulans* obtained from beef meat. Three of them were β-hemolytic and one α-hemolytic. In an assay of cytotoxic activity using VERO cells, all the isolates caused cell lysis. Three isolates produced slime and two were biofilm producers.

*S. lutrae* from otters has been reported to be catalase and coagulase positive; weakly DNase positive and hemolytic on sheep blood agar. Clumping factor and hyaluronidase are not produced (Foster et al. 1997).

## 4. Human health implications of these virulence factors

Humans can be affected by staphylococcal virulence factors in two main ways. Firstly, close contact with animals can facilitate interspecies transmission and adaptation of staphylococci from animals to humans. Secondly, indirect transmission can occur, by foods of animal origin or by devices contaminated with animal strains of staphylococci.

Staphylococci belonging to SIG are mainly skin and mucous membrane commensals of animals, especially companion animals. They might cause diseases, including otitis or skin infections, mostly when the immune status is compromised or barriers are broken. Infections in humans due to *S. pseudintermedius* have been described but the role of different virulence factors in human infection is scarcely studied. *In vitro*, *S. pseudintermedius* exhibits similar behavior to *S. aureus* towards human cells (Maali et al. 2018), harboring similar virulence factors, but human *S. pseudintermedius* infections are scarce, probably due to a very low carriage rate. Even in higher risk population groups (pet owners, veterinary staff, farmers) carriage of *S. pseudintermedius* seems to be uncommon but it is possible that misdiagnoses happen. In Sweden, 13 of 101 isolates of *S. aureus* from human samples of infected dog bite wounds were found to be in fact *S. pseudintermedius.* All isolates carried *LukF/S-I*, *siet* and *se-int* genes; two isolates carried *exp*A and one, *exp*B and *sec_canine_* (Börjesson et al. 2015). Similar results were found in a study of zoonotic transmission of *S. pseudintermedius* between dogs and healthy dog-owning household members (Gómez-Sanz et al. 2013). Screening CoP *Staphylococcus* nasal carriage in pet-owning households (Gómez-Sanz et al. 2013) revealed three owners (4.5%) to be colonized by *S. pseudintermedius*. One of the strains was identical to the one isolated from the corresponding pet. Two of the strains isolated from owners carried the exfoliative toxin gene *exp*A, reinforcing the idea that pet ownership could be a potential risk factor to acquire virulent bacteria.

A cluster of four cases of infections in a tertiary hospital caused by the same strain of MRSP was described (Starlander et al. 2014). Three of the patients had wound infections and *LukF/S-I* and *siet* genes were detected in the bacterial isolates. The animal source could not be identified.

*S. pseudintermedius* carrying exfoliative toxin *siet* and *LukF/S-I* genes was isolated from a husky dog and its owner suffering from a severe skin infection (Robb et al. 2017). Somayaji et al. (2016) had previously reported 24 cases of human infections due to this bacterial species but no investigation of virulence factors was done in their study.

Some sequence types of *S. pseudintermedius*, under *in vitro* conditions, seems to adhere better to human keratinocytes. Enhanced adherence of a human isolate of MRSP ST71 to human keratinocytes was observed (Latronico et al. 2014) and it has been suggested that this lineage of *S. pseudintermedius* could have a higher zoonotic potential than others. In Hong Kong, nasal carriage of MRSP ST71 was demonstrated in a veterinary nurse working with small animals (Boost et al. 2011).

Using human osteoblasts, Maali et al. (2016) demonstrated that *S. pseudintermedius* can adhere to human fibronectin and it has greater internalization ability, intracellular persistence and cytotoxicity than *S. aureus*.

Antibiotic-resistant biofilm formation has been described in a strain of *S. pseudintermedius* isolated from a wound of a human patient with chronic lymphoblastic leukemia (Pompilio et al. 2015). The patient had had close contact with a companion dog as well as cattle. The biofilm was resistant to many antibiotics, including linezolid, tigecycline and vancomycin.

Biofilm production, β-hemolysis and cytotoxic activity to Vero cells have been described in *S. hyicus* isolated from beef meat in Egypt (Osman et al. 2016). As previously mentioned, genes encoding enterotoxins *seb* and *sec*, and for toxic shock syndrome toxin *tst1*, in an isolate of *S. agnetis* obtained from small ruminant milk were observed (Rahmdel et al. 2018). In the same study, two strains of *S. pseudintermedius*, one of them carrying genes *sea*, *seb*, *sec*, *see* and *tst1* and the other, *sec* and *tst1* were found. Due to the low number of isolates included in these studies, the real importance of these findings is difficult to evaluate.

*S. pseudintermedius* isolated from a case of endocarditis in a human patient following implantation of a cardioverter-defibrillator device was proposed to be of canine origin (Riegel et al. 2011). The culture supernatant of the isolate showed leukotoxin activity against human neutrophils but it was not neutralized by anti-PVL antibodies suggesting that leukotoxic activity could not be attributed to PVL leukotoxin. Exfoliative toxin was not detected in this isolate.

Recently, the first case of human infection due to *S. delphini* has been described (Magleby et al. 2019), but virulence factors that might be present were not studied.

## 5. Conclusions

Recent studies on virulence factors in coagulase-positive Staphylococci of veterinary interest other than *S. aureus* are reviewed in this paper and a summary is presented in Table 1. Most of the literature concerns *S. pseudintermedius*, whose virulence factors closely resemble those of *S. aureus.* In many cases, virulence factors observed are the same in isolates from healthy and diseased animals, reinforcing the idea that some host factors also play a significant role in the development and outcome of the disease.

**Table 1.** Summary of the main virulence factors described in coagulase positive Staphylococci other than *S. aureus*.

Virulence factor	Method*	*S. pseudintermedius*	*S. intermedius*	*S. delphini*	*S. hyicus*	*S. agnetis*	*S. schleiferi*
Surface protein							
Protein A	Gene Spa**				*+ [63]*		
	Gene SpsA***	*+ [13]*					
	Dot-blot	*+ [30]*					
	Agglutination		*+ [28]*				
Protein B and C	Gene *SpsB*, SpsC***	*+ [13]*					
Protein D	Gene Sps D**	*+ [55][21]*					
	Gene Sps D***	*+ [10] [13]*					
Protein Eto I	Gene *SpsE* to SpsI***	*+ [13]*					
Protein K	Gene SpsK***	*+ [13]*					
Protein L	Gene Sps L**	*+ [55] [21]*					
	Gene Sps***	*[13]*					
Protein M and N	Gene *SpsM*, SpsN***	*+ [13]*					
Protein O	Gene Sps O***	*+ [10][13]*					
	Gene Sps O**	*+ [21]*					
Protein P	Gene Sps P**	*+ [9]*					
	Gene Sps P***	*+ [13]*					
Protein Q	Gene Sps Q**	*+ [7] [9]*					
	Gene SpsQ***	*+ [13]*					
Protein R	Gene SpsR**	*+ [9]*					
Elastin binding protein	Gene ebpS**		*+ [67]*				
	Gene ebp***					*+ [4]*	
Laminin binding protein	Gene eno**		*+ [67]*				
Fibrinogen binding protein	Gene fib**		*+ [67]*				
	Gene fib***					*+ [6]*	
	Genes associated***					*+ [58]*	
Clumping factor /	Agglutination		*+ [28]*				
Fibronectin binding protein A	Gene fnbA**		*+ [67]*				
	Gene fnbA***					*+ [4]*	
	Genes associated***					*+ [58]*	
Fibronectin binding protein B	Gene fnbB***					*+ [4]*	
	Genes associated***					*+ [58]*	
Immunoglobulin-binding protein (sbi)	Gene sbi***					*+ [6]*	
Von Willebrand factor binding protein	Gene vwb***					*+ [6]*	
Collagen adhesin	Gene can***					*+ [4]*	
Biofilm							
Biofilm formation activity	Absorbance	*+ [82][20][74][12][25] [57]*	*+ [28][82]*	*+ [77]*			
	Congo red agar	*+ [20]*	*+ [54]*		*+ [54]*		*+ [54]*
	Tube adherence test	*+ [20][21]*	*+ [54]*		*+ [54]*		*+ [54]*
Biofilm associated protein	Gene bap**		*+ [67]*				
Biofilm associated polysaccharide	Gene icaA**	*+ [20][74]*					
	Gene icaA***	*+ [13]*					
	Gene icaB***	*+ [13]*					
	Gene icaC***	*+ [13]*					
	Gene icaD**	*+ [20][74]*					
	Operon Ica**	*+ [21]*					
Phenol-Soluble Modulins PSM							
δ-toxin	Gene hld***				*+ [19]*		
	Gene hld**	*+ [47]*					
PSMε	Gene psmε**	*+ [47]*					
PSM-mec	Gene psm-mec***	*+ [13]*					
Hemolysis							
Hemolytic activity	Microplate method		*+ [28]*				
α-hemolysis activity							*+ [54]*
β -hemolysis activity					*+ [54]*		*+ [54]*
γ-hemolysin	Gene hlg γ**	*+ [56][35][32]*				*+ [50]*	
β-hemolysin	Gene hlb***	*+ [13]*				*+ [6][4]*	
	Genes associated***					*+ [18]*	
α-hemolysin	Gene hla**	*+ [32][36]*					
δ-hemolysin	Gene hld**	*+ [36]*					
Hemolysin III	Genes associated***					*+ [6]*	
Enzymes							
Protease	Activity on casein agar plates		*+ [28]*				
Protease Clp P	Gene clpP***	*+ [13]*					
Protease Clp X	Gene clpX***	*+ [13]*					
Arginine catabolic mobile element (ACME)	Gene arcA**	*+ [84]*					
Nuclease C	Gene nuc***	*+ [13]*				*+ [6][4]*	
Surface-associated glycosidases (NanB)	Gene nan B***	*+ [13]*					
Aromatic aminoacid decarboxylase (SadA)	Gene sad A***	*+ [13]*					
Epidermal cell differentiation inhibitor toxin homolog (EDIN)	Genes associated***				*+ [19][46]*		
Hyaluronidase	Genes associated***					*+ [18]*	
	Gene hysA***					*+ [6][4]*	
Phosphatidyl glycerol lysyl transferase	Gene *mprF* (fmtC)***					*+ [6]*	
Hemeoxygenases staphylobilin-producing	Gene isdl***					*+ [6]*	
Sortases A	Gene srtA***					*+ [6]*	
Glyceraldehyde 3-phosphate dehydrogenase	Gene *gapA1*, gapA2***					*+ [6]*	
Zinc metalloproteinase aureolysin	Gene aur***					*+ [6]*	
Staphopain A (Cysteine proteinase A)	Gene *sspP* (scpA)***					*+ [6][4]*	
Enterotoxins							
SEA	Gene sea**	*+ [56][79][78][32][34]*	*+ [67]*				
SEB	Gene seb**	*+ [32]*	*+ [67]*			*+ [59]*	
SEC	Gene sec**	*+ [56] [79][32]*	*+ [67]*			*+ [59]*	
SED	Gene sed**	*+ [32]*	*+ [67]*				
SEE	Gene see**		*+ [67]*				
SEH	Gene she**	*+ [21][36]*					
SEI	Gene sei**	*+ [32]*					
SEJ	Gene sej**	*+ [32]*					
SEK	Gene seK**	*+ [32]*					
SEL	Gene *sell*, Gene selq**	*+ [79][17][36]*					
	Gene sel**	*+ [33][34]*					
SER	Gene ser**	*+ [32]*					
SET	Gene set7***					*+ [6]*	
	Gene *set15*, *set16*, set34***					*+ [4]*	
SEC_canine_	Gene *secc* < nine**	*+ [30][85][86][33] [21][32][34][17][36]*		*+ [31]*			
SE-int	Gene se-int**	*+ [79][35][33] [21] [32] [34][17][36]*		*+ [31][65]*			
	Gene se-int***	*+ [13]*					
Enterotoxins, superantigen	Genes associated***					*+ [58]*	
Toxic shock syndrome toxin	Gene tst**	*+ [30][56][79]*				*+ [59]*	
Exfoliative toxin							
ETA	Genes associated***					*+ [6][4]*	
ETD	Gene etd**					*+ [50]*	
EXI / ExpA	Gene exi/ expA**	*+ [29][39][56] [33][78][32][34][17]*		*+ [31]*			
ExpB	Gene expB**	*+ [40][33][21][78][17]*					
SIET	Gene siet**	*+ [30][12][66] [85][86][56][42][35] [33][21][78][25][32] [34] [17][36][62]*	*+ [86]*	*+ [31][65]*			
	Gene siet***	*+ [13]*					
SPETA (SHETA)	Gene *speta*, Gene sheta**	*+ [21]*			*+ [41]*		
	Gene *speta*, Gene sheta***	*+ [13]*					
	Chromatography				*+ [5]*		
	Western blot, Dot blot hybridization				*+ [70]*		
SHETB	Western blot Dot blot hybridization				*+ [70]*		
	Gene shetb****				*+ [83]*		
ExhA, ExhB, ExhC, and ExhD	Gene *ExhA*, *ExhB*, *ExhC*, and ExhD****				*+ [2]*		
	Gene *ExhA*, *ExhB*, *ExhC*, and ExhD*****				*+ [53]*		
	Gene *ExhA, ExhB, ExhC*, and ExhD**				*+ [27]*		
	Gene *ExhA*, *ExhB*, *ExhC*, and *ExhD*:***				*+ [46]*		
ExhA,	Gene ExhA***				*+ [19]*		
Leukocidin							
Luk S/F-I or Luk-I	Gene *lukS-I* and/or *luk F-*I**	*+ [66][56][47][35][33][21][32][34] [17][36][ 62]*		*+ [31][65]*			
	Gene *lukS-I* and/or lukF-I***	*+ [13]*					
	Spectrometry	*+ [1]*					
Global regulatory elements System saeRS	Gene *sae S* / Gene saeR***	*+ [13]*					
	Gene *capA, capB, capC, capD, capE, capF, capG, cap8H, cap8I, cap8J, cap8K, capL, capM, capP, capO, cap1A*:***					*+ [4]*	

* Method used to detect or to describe virulence factor.

** Gene detected by PCR.

*** Gene detected by Genome Sequence Analysis.

**** Gene detected by Cloning and expression.

***** Review.

The effect of these virulence factors on human health is also described. The possibility of misdiagnosis of species of CoPS has been demonstrated in human clinical samples. Prevalence of zoonotic infections could be higher than thought and medical claboratories should be aware of these other staphylococcal species. In keeping with the One Health approach to animal and human disease, medical professionals, veterinarians and health workers should be aware of the risks derived from exposure to these bacteria in people in close contact with animals, including pet owners, farmers and veterinarians themselves.

## References

[CIT0001] Abouelkhair MA, Bemis DA, Giannone RJ, Frank LA, Kania SA. 2018. Characterization of a leukocidin identified in *Staphylococcus pseudintermedius*. PLoS One. 13(9):e0204450. 30261001 10.1371/journal.pone.0204450 PMC6160070

[CIT0002] Ahrens P, Andresen LO. 2004. Cloning and sequence analysis of genes encoding *Staphylococcus hyicus* exfoliative toxin types A, B, C, and D. J Bacteriol. 186(6):1833–1837. 14996814 10.1128/JB.186.6.1833-1837.2004 PMC355961

[CIT0003] Aires-de-Sousa M. 2017. Methicillin-resistant *Staphylococcus aureus* among animals: current overview. Clin Microbiol Infect. 23(6):373–380. 27851997 10.1016/j.cmi.2016.11.002

[CIT0004] Al-Rubaye AAK, Couger MB, Ojha S, Pummill JF, Koon JA, Wideman RF, Rhoads DD. 2015. Genome analysis of *Staphylococcus agnetis*, an agent of lameness in broiler chickens. PLoS One. 10(11):e0143336. 26606420 10.1371/journal.pone.0143336 PMC4659636

[CIT0005] Andresen LO, Bille-Hansen V, Wegener HC. 1997. *Staphylococcus hyicus* exfoliative toxin: Purification and demonstration of antigenic diversity among toxins from virulent strains. Microb Pathog. 22(2):113–122. 9050000 10.1006/mpat.1996.0097

[CIT0006] Åvall-Jääskeläinen S, Taponen S, Kant R, Paulin L, Blom J, Palva A, Koort J. 2018. Comparative genome analysis of 24 bovine-associated *Staphylococcus* isolates with special focus on the putative virulence genes. Peer J. 6:e4560. 29610707 10.7717/peerj.4560 PMC5880176

[CIT0007] Balachandran M, Bemis DA, Kania SA. 2018. Expression and function of protein a in *Staphylococcus pseudintermedius*. Virulence. 9(1):390–401. 29157101 10.1080/21505594.2017.1403710 PMC5955199

[CIT0008] Ballhausen B, Kriegeskorte A, van Alen S, Jung P, Köck R, Peters G, Bischoff M, Becker K. 2017. The pathogenicity and host adaptation of livestock-associated MRSA CC398. Vet Microbiol. 200:39–45. 27236228 10.1016/j.vetmic.2016.05.006

[CIT0009] Bannoehr J, Ben Zakour NL, Reglinski M, Inglis NF, Prabhakaran S, Fossum E, Smith DG, Wilson GJ, Cartwright RA, Haas J, et al. 2011. Genomic and surface proteomic analysis of the canine pathogen staphylococcus pseudintermedius reveals proteins that mediate adherence to the extracellular matrix. Infect Immun. 79(8):3074–3086. 10.1128/IAI.00137-11. 21576333 PMC3147560

[CIT0010] Bannoehr J, Ben Zakour NL, Waller AS, Guardabassi L, Thoday KL, Van Den Broek AHM, Fitzgerald JR. 2007. Population genetic structure of the *Staphylococcus intermedius* group: Insights into agr diversification and the emergence of methicillin-resistant strains. J Bacteriol. 189(23):8685–8692. 17905991 10.1128/JB.01150-07 PMC2168937

[CIT0011] Bannoehr J, Brown JK, Shaw DJ, Fitzgerald RJ, van den Broek AHM, Thoday KL. 2012. *Staphylococccus pseudintermedius* surface proteins SpsD and SpsO mediate adherence to ex vivo canine corneocytes. Vet Dermatol. 23(2):119–e26. 22112246 10.1111/j.1365-3164.2011.01021.x

[CIT0012] Bardiau M, Yamazaki K, Ote I, Misawa N, Mainil JG. 2013. Characterization of methicillin-resistant *Staphylococcus pseudintermedius* isolated from dogs and cats. Microbiol Immunol. 57 (7):n/a–501. 10.1111/1348-0421.12059 23607810

[CIT0013] Bergot M, Martins-Simoes P, Kilian H, Châtre P, Worthing KA, Norris JM, Madec JY, Laurent F, Haenni M. 2018. Evolution of the population structure of *Staphylococcus pseudintermedius* in France. Front Microbiol. 9: 1–10. 30619143 10.3389/fmicb.2018.03055 PMC6300469

[CIT0014] Blaiotta G, Fusco V, Ercolini D, Pepe O, Coppola S. 2010. Diversity of *Staphylococcus* species strains based on partial kat (catalase) gene sequences and design of a PCR-restriction fragment length polymorphism assay for identification and differentiation of coagulase-positive species (*S. aureus*, *S. delphini*, *S. hyicus*, *S. intermedius*, *S. pseudintermedius* and *S. scheleiferi* subsp. *coagulans*. ). J Clin Microbiol. 48(1):192–201. 19889901 10.1128/JCM.00542-09 PMC2812296

[CIT0015] Bond R, Loeffler A. 2012. What’s happened to *Staphylococcus intermedius*? Taxonomic revision and emergence of multi-drug resistance. J Small Anim Pract. 53(3):147–154. 22251285 10.1111/j.1748-5827.2011.01165.x

[CIT0016] Boost MV, So SYC, Perreten V. 2011. Low rate of methicillin-resistant coagulase-positive staphylococcal colonization of veterinary personnel in Hong Kong. Zoonoses Public Health. 58(1):36–40. 19968853 10.1111/j.1863-2378.2009.01286.x

[CIT0017] Börjesson S, Gómez-Sanz E, Ekström K, Torres C, Grönlund U. 2015. *Staphylococcus pseudintermedius* can be misdiagnosed as *Staphylococcus aureus* in humans with dog bite wounds. Eur J Clin Microbiol Infect Dis. 34(4):839–844. 25532507 10.1007/s10096-014-2300-y

[CIT0018] Calcutt MJ, Foecking MF, Fry PR, Hsieh H-Y, Perry J, Stewart GC, Scholl DT, Messier S, Middleton JR. 2014. Draft Genome Sequence of Bovine Mastitis Isolate *Staphylococcus agnetis* CBMRN 20813338. Genome Announc. 2(5): 1–2. 10.1128/genomeA.00883-14 PMC4155595 25189590

[CIT0019] Calcutt MJ, Foecking MF, Hsieh H-Y, Adkins PRF, Stewart GC, Middleton JR. 2015. Sequence Analysis of *Staphylococcus hyicus* ATCC 11249 T, an etiological agent of exudative epidermitis in Swine, reveals a Type VII secretion system locus and a novel 116-kilobase genomic island harboring toxin-encoding genes. Genome Announc. 3(1): 1–2. 10.1128/genomeA.01525-14 PMC4335327 25700402

[CIT0020] Casagrande Proietti P, Stefanetti V, Hyatt DR, Marenzoni ML, Capomaccio S, Coletti M, Bietta A, Franciosini MP, Passamonti F. 2015. Phenotypic and genotypic characterization of canine pyoderma isolates of *Staphylococcus pseudintermedius* for biofilm formation. J Vet Med Sci. 77(8):945–951. 25892615 10.1292/jvms.15-0043 PMC4565817

[CIT0021] Couto N, Belas A, Oliveira M, Almeida P, Clemente C, Pomba C. 2016. Comparative RNA-seq-based transcriptome analysis of the virulence characteristics of methicillin-resistant and -susceptible *Staphylococcus pseudintermedius* strains isolated from small animals. Antimicrob Agents Chemother. 60(2):962–967. 26621622 10.1128/AAC.01907-15 PMC4750686

[CIT0022] De La Fuente R, Suarez G, Schleifer KH. 1985. *Staphylococcus aureus* subsp. *anaerobius* subsp. nov., the Causal Agent of Abscess Disease of Sheep. Int J Syst Bacteriol. 35(1):99–102.

[CIT0023] Decristophoris P, Fasola A, Benagli C, Tonolla M, Petrini O. 2011. Identification of *Staphylococcus intermedius* Group by MALDI-TOF MS. Syst Appl Microbiol. 34(1):45–51. 21300509 10.1016/j.syapm.2010.11.004

[CIT0024] Devriese LA, Hajek V, Oeding P, Meyer SA, Schleifer KH. 1978. *Staphylococcus hyicus* (Sompolinsky 1953) comb. nov. and *Staphylococcus hyicus* subsp. *chromogenes* subsp. nov. Int J Syst Bacteriol. 28(4):482–490.

[CIT0025] Devriese LA, Vancanneyt M, Baele M, Vaneechoutte M, De Graef E, Snauwaert C, Cleenwerck I, Dawyndt P, Swings J, Decostere A, et al. 2005. *Staphylococcus pseudintermedius* sp. nov., a coagulase-positive species from animals. Int J Syst Evol Microbiol. 55(4):1569–1573. 16014483 10.1099/ijs.0.63413-0

[CIT0026] Diribe O, Thomas S, AbuOun M, Fitzpatrick N, La Ragione R. 2015. Genotypic relatedness and characterization of *Staphylococcus pseudintermedius* associated with post-operative surgical infections in dogs. J Med Microbiol. 64(9):1074–1081. 26449388 10.1099/jmm.0.000110

[CIT0027] Foster G, Ross HM, Hutson RA, Collins MD. 1997. *Staphylococcus lutrae* sp. nov., a new coagulase-positive species isolated from Otters. Int J Syst Bacteriol. 47(3):724–726. 9226903 10.1099/00207713-47-3-724

[CIT0028] Futagawa-Saito K, Ba-Thein W, Higuchi T, Sakurai N, Fukuyasu T. 2007. Nationwide molecular surveillance of exfoliative toxigenic *Staphylococcus hyicus* on pig farms across Japan. Vet Microbiol. 124(3–4):370–374. 17543479 10.1016/j.vetmic.2007.04.036

[CIT0029] Futagawa-Saito K, Ba-Thein W, Sakurai N, Fukuyasu1 T. 2006. Prevalence of virulence factors in *Staphylococcus intermedius* isolates from dogs and pigeons. BMC Vet Res [Internet]. 2(4):1–5. http://www.biomedcentral.com/1746-6148/2/4. 10.1186/1746-6148-2-4 PMC1403770 16438708

[CIT0030] Futagawa-Saito K, Makino S, Sunaga F, Kato Y, Sakurai-Komada N, Ba-Thein W, Fukuyasu T. 2009. Identification of first exfoliative toxin in *Staphylococcus pseudintermedius*. FEMS Microbiol Lett. 301(2):176–180. 19891731 10.1111/j.1574-6968.2009.01823.x

[CIT0031] Garbacz K, Żarnowska S, Piechowicz L, Haras K. 2013. Pathogenicity potential of *Staphylococcus pseudintermedius* strains isolated from canine carriers and from dogs with infection signs. Virulence. 4(3):255–259. 23328490 10.4161/viru.23526 PMC3711984

[CIT0032] Gharsa H, Ben Slama K, Gómez-Sanz E, Lozano C, Klibi N, Jouini A, Messadi L, Boudabous A, Torres C. 2013. Antimicrobial resistance, virulence genes, and genetic lineages of *Staphylococcus pseudintermedius* in healthy dogs in Tunisia. Microb Ecol. 66(2):363–368. 23686400 10.1007/s00248-013-0243-y

[CIT0033] Gharsa H, Slama KB, Gómez-Sanz E, Gómez P, Klibi N, Zarazaga M, Boudabous A, Torres C. 2015. Characterisation of nasal *Staphylococcus delphini* and *Staphylococcus pseudintermedius* isolates from healthy donkeys in Tunisia. Equine Vet J. 47(4):463–466. 24913693 10.1111/evj.12305

[CIT0034] Gómez-Sanz E, Torres C, Benito D, Lozano C, Zarazaga M. 2013. Animal and human *Staphylococcus aureus* associated clonal lineages and high rate of *Staphylococcus pseudintermedius* novel lineages in Spanish kennel dogs: Predominance of S. aureus ST398. Vet Microbiol. 166(3–4):580–589. 23932077 10.1016/j.vetmic.2013.07.014

[CIT0035] Gómez-Sanz E, Torres C, Ceballos S, Lozano C, Zarazaga M. 2013. Clonal Dynamics of Nasal *Staphylococcus aureus* and *Staphylococcus pseudintermedius* in Dog-Owning Household Members. Detection of MSSA ST398. PLoS One. 8(7):e69337. 23874949 10.1371/journal.pone.0069337 PMC3706376

[CIT0036] Gómez-Sanz E, Torres C, Lozano C, Sáenz Y, Zarazaga M. 2011. Detection and characterization of methicillin-resistant *Staphylococcus pseudintermedius* in healthy dogs in La Rioja, Spain. Comp Immunol Microbiol Infect Dis. 34(5):447–453. 21903268 10.1016/j.cimid.2011.08.002

[CIT0037] Gómez-Sanz E, Torres C, Lozano C, Zarazaga M. 2013. High diversity of *Staphylococcus aureus* and *Staphylococcus pseudintermedius* lineages and toxigenic traits in healthy pet-owning household members. Underestimating normal household contact? Comp Immunol Microbiol Infect Dis. 36(1):83–94. 23153600 10.1016/j.cimid.2012.10.001

[CIT0038] Hajek V. 1976. *Staphylococcus intermedius*, a New Species Isolated from Animals. Int J Syst Bacteriol. 26(4):401–408.

[CIT0039] Igimi S, Takahashi E, Mitsuoka T. 1990. *Staphylococcus schleiferi* subsp. *coagulans* subsp. nov., isolated from the external auditory meatus of dogs with external ear otitis. Int J Syst Bacteriol. 40(4):409–411. 2275856 10.1099/00207713-40-4-409

[CIT0040] Iyori K, Futagawa-Saito K, Hisatsune J, Yamamoto M, Sekiguchi M, Ide K, Son W-G, Olivry T, Sugai M, Fukuyasu T, et al. 2011. *Staphylococcus pseudintermedius* exfoliative toxin EXI selectively digests canine desmoglein 1 and causes subcorneal clefts in canine epidermis. Vet Dermatol. 22(4):319–326. 21410798 10.1111/j.1365-3164.2011.00952.x

[CIT0041] Iyori K, Hisatsune J, Kawakami T, Shibata S, Murayama N, Ide K, Nagata M, Fukata T, Iwasaki T, Oshima K, et al. 2010. Identification of a novel *Staphylococcus pseudintermedius* exfoliative toxin gene and its prevalence in isolates from canines with pyoderma and healthy dogs. FEMS Microbiol Lett. 312(2):169–175. 20875053 10.1111/j.1574-6968.2010.02113.x

[CIT0042] Kanbar T, Voytenko AV, Alber J, Lämmler C, Weiss R, Skvortzov VN. 2008. Distribution of the putative virulence factor encoding gene sheta in *Staphylococcus hyicus* strains of various origins. J Vet Sci. 9(3):327–329. 18716454 10.4142/jvs.2008.9.3.327 PMC2811846

[CIT0043] Kang MH, Chae MJ, Yoon JW, Kim SG, Lee SY, Yoo JH, Park HM. 2014. Antibiotic resistance and molecular characterization of ophthalmic *Staphylococcus pseudintermedius* isolates from dogs. J Vet Sci. 15(3):409–415. 24690601 10.4142/jvs.2014.15.3.409 PMC4178142

[CIT0044] Kong C, Neoh HM, Nathan S. 2016. Targeting *Staphylococcus aureus* toxins: A potential form of anti-virulence therapy. Toxins (Basel). 8(3):72. 10.3390/toxins8030072 PMC4810217 26999200

[CIT0045] Latronico F, Moodley A, Nielsen SS, Guardabassi L. 2014. Enhanced adherence of methicillin-resistant *Staphylococcus pseudintermedius* sequence type 71 to canine and human corneocytes. Vet Res. 45(1):70. 24957656 10.1186/1297-9716-45-70 PMC4087241

[CIT0046] Leekitcharoenphon P, Pamp SJ, Andresen LO, Aarestrup FM. 2016. Comparative genomics of toxigenic and non-toxigenic *Staphylococcus hyicus*. Vet Microbiol. 185:34–40. 26931389 10.1016/j.vetmic.2016.01.018

[CIT0047] Maali Y, Badiou C, Martins-Simões P, Hodille E, Bes M, Vandenesch F, Lina G, Diot A, Laurent F, Trouillet-Assant S. 2018. Understanding the Virulence of *Staphylococcus pseudintermedius*: A major role of pore-forming toxins. Front Cell Infect Microbiol. 8: 1–10. 30003063 10.3389/fcimb.2018.00221 PMC6032551

[CIT0048] Maali Y, Martins-Simões P, Valour F, Bouvard D, Rasigade JP, Bes M, Haenni M, Ferry T, Laurent F, Trouillet-Assant S. 2016. Pathophysiological mechanisms of *Staphylococcus* non-aureus bone and joint infection: Interspecies homogeneity and specific behavior of *S*. pseudintermedius. Front Microbiol. 7: 1–9. 27462303 10.3389/fmicb.2016.01063 PMC4940379

[CIT0049] Magleby R, Bemis DA, Kim D, Carroll KC, Castanheira M, Kania SA, Jenkins SG, Westblade LF. 2019. First reported human isolation of *Staphylococcus delphini*. Diagn Microbiol Infect Dis. 94(3):274–276. 30955895 10.1016/j.diagmicrobio.2019.01.014

[CIT0050] Mahato S, Mistry HU, Chakraborty S, Sharma P, Saravanan R, Bhandari V. 2017. Identification of variable traits among the methicillin resistant and sensitive coagulase negative staphylococci in milk samples from mastitic cows in India. Front Microbiol. 8: 1–7. 28824577 10.3389/fmicb.2017.01446 PMC5534481

[CIT0051] Murray AK, Lee J, Bendall R, Zhang L, Sunde M, Slettemeås JS, Gaze W, Page AJ, Vos M. 2018. *Staphylococcus cornubiensis* sp. nov., a member of the *Staphylococcus intermedius* group (SIG). Int J Syst Evol Microbiol. 68(11):3404–3408. 30204583 10.1099/ijsem.0.002992

[CIT0052] Mutters NT, Bieber CP, Hauck C, Reiner G, Malek V, Frank U. 2016. Comparison of livestock-associated and health care–associated MRSA—genes, virulence, and resistance. Diagn Microbiol Infect Dis. 86(4):417–421. 27640079 10.1016/j.diagmicrobio.2016.08.016

[CIT0053] Nishifuji K, Sugai M, Amagai M. 2008. Staphylococcal exfoliative toxins: ‘Molecular scissors’ of bacteria that attack the cutaneous defense barrier in mammals. J Dermatol Sci. 49(1):21–31. 17582744 10.1016/j.jdermsci.2007.05.007

[CIT0054] Osman KM, Amer AM, Badr JM, Helmy NM, Elhelw RA, Orabi A, Bakry M, Saad A. 2016. Antimicrobial resistance, biofilm formation and mecA characterization of methicillin-susceptible *S. aureus* and non-*S. aureus* of beef meat origin in Egypt. Front Microbiol. 7: 1–12. 26973606 10.3389/fmicb.2016.00222 PMC4770614

[CIT0055] Pietrocola G, Gianotti V, Richards A, Nobile G, Geoghegan JA, Rindi S, Monk IR, Bordt AS, Foster TJ, Fitzgerald JR, et al. 2015. Fibronectin binding proteins SpsD and SpsL both support invasion of canine epithelial cells by *Staphylococcus pseudintermedius*. Infect Immun. 83(10):4093–4102. 26238710 10.1128/IAI.00542-15 PMC4567651

[CIT0056] Pitchenin LC, Brandão LNS, Rosa JMA, Kagueyama FC, Alves A da S, Rocha ÍSM, Nakazato L, Dutra V. 2018. Occurrence of toxin genes in *Staphylococcus pseudintermedius* from diseased dogs and other domestic and wild species. J Infect Dev Ctries. 11(12):957–961. 31626602 10.3855/jidc.8261

[CIT0057] Pompilio A, De Nicola S, Crocetta V, Guarnieri S, Savini V, Carretto E, Di Bonaventura G. 2015. New insights in *Staphylococcus pseudintermedius* pathogenicity: Antibiotic-resistant biofilm formation by a human wound-associated strain. BMC Microbiol. 15(1): 1–14. 25994406 10.1186/s12866-015-0449-x PMC4440327

[CIT0058] Poulsen LL, Thøfner I, Bisgaard M, Olsen RH, Christensen JP, Christensen H. 2017. *Staphylococcus agnetis*, a potential pathogen in broiler breeders. Vet Microbiol. 212:1–6. 29173582 10.1016/j.vetmic.2017.10.018

[CIT0059] Rahmdel S, Hosseinzadeh S, Shekarforoush SS, Torriani S, Gatto V, Pashangeh S. 2018. Safety hazards in bacteriocinogenic *Staphylococcus* strains isolated from goat and sheep milk. Microb Pathog. 116:100–108. 29355699 10.1016/j.micpath.2018.01.016

[CIT0060] Rainard P, Foucras G, Fitzgerald JR, Watts JL, Koop G, Middleton JR. 2018. Knowledge gaps and research priorities in *Staphylococcus aureus* mastitis control. Transbound Emerg Dis. 65:149–165. 28984427 10.1111/tbed.12698

[CIT0061] Riegel P, Jesel-Morel L, Laventie B, Boisset S, Vandenesch F, Prévost G. 2011. Coagulase-positive *Staphylococcus pseudintermedius* from animals causing human endocarditis. Int J Med Microbiol. 301(3):237–239. 21075051 10.1016/j.ijmm.2010.09.001

[CIT0062] Robb AR, Wright ED, Foster AME, Walker R, Malone C. 2017. Skin infection caused by a novel strain of *Staphylococcus pseudintermedius* in a Siberian husky dog owner. JMM Case Reports. 4(3): 1–4. 10.1099/jmmcr.0.005087 PMC5382809 28663821

[CIT0063] Rosander A, Guss B, Pringle M. 2011. An IgG-binding protein A homolog in *Staphylococcus hyicus*. Vet Microbiol. 149(1-2):273–276. 21111546 10.1016/j.vetmic.2010.10.011

[CIT0064] Ross Fitzgerald J. 2009. The *Staphylococcus intermedius* group of bacterial pathogens: Species re-classification, pathogenesis and the emergence of meticillin resistance. Vet Dermatol. 20(5–6):490–495. 20178486 10.1111/j.1365-3164.2009.00828.x

[CIT0065] Ruiz-Ripa L, Gómez P, Alonso CA, Camacho MC, de la Puente J, Fernández-Fernández R, Ramiro Y, Quevedo MA, Blanco JM, Zarazaga M, et al. 2019. Detection of MRSA of Lineages CC130-mecC and CC398-mecA and *Staphylococcus delphini*-lnu(A) in Magpies and Cinereous Vultures in Spain. Microb Ecol. 78(2):409–415. 30694341 10.1007/s00248-019-01328-4

[CIT0066] Ruzauskas M, Couto N, Pavilonis A, Klimiene I, Siugzdiniene R, Virgailis M, Vaskeviciute L, Anskiene L, Pomba C. 2016. Characterization of *Staphylococcus pseudintermedius* isolated from diseased dogs in Lithuania. Pol J Vet Sci. 19(1):7–14. 27096782 10.1515/pjvs-2016-0002

[CIT0067] Salaberry SRS, Saidenberg ABS, Zuniga E, Melville PA, Santos FGB, Guimarães EC, Gregori F, Benites NR. 2015. Virulence factors genes of *Staphylococcus* spp. isolated from caprine subclinical mastitis. Microb Pathog. 85:35–39. 26026835 10.1016/j.micpath.2015.05.007

[CIT0068] Sasaki T, Kikuchi K, Tanaka Y, Takahashi N, Kamata S, Hiramatsu K. 2007. Reclassification of phenotypically identified *Staphylococcus intermedius* strains. J Clin Microbiol. 45(9):2770–2778. 17596353 10.1128/JCM.00360-07 PMC2045239

[CIT0069] Sasaki T, Tsubakishita S, Tanaka Y, Sakusabe A, Ohtsuka M, Hirotaki S, Kawakami T, Fukata T, Hiramatsu K. 2010. Multiplex-PCR method for species identification of coagulase-positive staphylococci. J Clin Microbiol. 48(3):765–769. 20053855 10.1128/JCM.01232-09 PMC2832457

[CIT0070] Sato H, Watanabe T, Higuchi K, Teruya K, Ohtake A, Murata Y, Saito H, Aizawa C, Danbara H, Maehara N. 2000. Chromosomal and extrachromosomal synthesis of exfoliative toxin from *Staphylococcus hyicus*. J Bacteriol. 182(14):4096–4100. 10869090 10.1128/jb.182.14.4096-4100.2000 PMC94597

[CIT0071] Scali F, Camussone C, Calvinho LF, Cipolla M, Zecconi A. 2015. Which are important targets in development of *S. aureus* mastitis vaccine? Res Vet Sci. 100:88–99. 25975626 10.1016/j.rvsc.2015.03.019

[CIT0072] Schmidt V, Nuttall T, Fazakerley J, McEwan N. 2009. *Staphylococcus intermedius* binding to immobilized fibrinogen, fibronectin and cytokeratin in vitro. Vet Dermatol. 20(5-6):502–508. 20178488 10.1111/j.1365-3164.2009.00804.x

[CIT0073] Seilie ES, Bubeck Wardenburg J. 2017. *Staphylococcus aureus* pore-forming toxins: The interface of pathogen and host complexity. Semin Cell Dev Biol. 72:101–116. 28445785 10.1016/j.semcdb.2017.04.003 PMC5823538

[CIT0074] Singh A, Walker M, Rousseau J, Weese JS. 2013. Characterization of the biofilm forming ability of *Staphylococcus pseudintermedius* from dogs. BMC Vet Res. 9(1):93. 23641755 10.1186/1746-6148-9-93 PMC3681638

[CIT0075] Somayaji R, Priyantha MAR, Rubin JE, Church D. 2016. Human infections due to *Staphylococcus pseudintermedius*, an emerging zoonosis of canine origin: report of 24 cases. Diagn Microbiol Infect Dis. 85(4):471–476. 27241371 10.1016/j.diagmicrobio.2016.05.008

[CIT0076] Starlander G, Borjesson S, Gronlund-Andersson U, Tellgren-Roth C, Melhus A. 2014. Cluster of infections caused by methicillin-resistant *Staphylococcus pseudintermedius* in humans in a tertiary hospital. J Clin Microbiol. 52(8):3118–3120. 24871217 10.1128/JCM.00703-14 PMC4136194

[CIT0077] Sudagidan M, Aydin A. 2012. Virulence properties of *Staphylococcus delphini* strains isolated from domestic pigeons. Med Weter [Internet]. 68 (4):231–236.

[CIT0078] Tabatabaei S, Najafifar A, Askari Badouei M, Zahraei Salehi T, Ashrafi Tamai I, Khaksar E, Abbassi MS, Ghazisaeedi F. 2019. Genetic characterisation of methicillin-resistant *Staphylococcus aureus* and *Staphylococcus pseudintermedius* in pets and veterinary personnel in Iran: new insights into emerging methicillin-resistant *S. pseudintermedius* (MRSP. ). J Glob Antimicrob Resist. 16:6–10. 30172831 10.1016/j.jgar.2018.08.022

[CIT0079] Tanabe T, Toyoguchi M, Hirano F, Chiba M, Onuma K, Sato H. 2013. Prevalence of staphylococcal enterotoxins in *Staphylococcus pseudintermedius* isolates from dogs with pyoderma and healthy dogs. Microbiol Immunol. 57(9):n/a–654. 10.1111/1348-0421.12069 23659343

[CIT0080] Taponen S, Supré K, Piessens V, van Coillie E, de Vliegher S, Koort J. 2012. *Staphylococcus agnetis* sp. nov., a coagulase variable species from bovine subclinical and mild clinical mastitis. Int J Syst Evol Microbiol. 62(1):61–65. 21335502 10.1099/ijs.0.028365-0

[CIT0081] Varaldo PE, Kilpper-Balz R, Biavasco F, Satta G, Schleifer KH. 1988. *Staphylococcus delphini* sp. nov., a Coagulase-Positive Species Isolated from Dolphins. Int J Syst Bacteriol. 38(4):436–439.

[CIT0082] Velázquez-Guadarrama N, Olivares-Cervantes AL, Salinas E, Martínez L, Escorcia M, Oropeza R, Rosas I. 2017. Presence of environmental coagulase-positive staphylococci, their clonal relationship, resistance factors and ability to form biofilm. Rev Argent Microbiol. 49(1):15–23. 28017482 10.1016/j.ram.2016.08.006

[CIT0083] Watanabe T, Sato H, Hatakeyama Y, Matsuzawa T, Kawai M, Aizawa C, Danbara H, Maehara N. 2000. Cloning of the gene coding for *Staphylococcus intermedius* exfoliative toxin and its expression in Escherichia coli. J Bacteriol. 182(14):4101–4103. 10869091 10.1128/jb.182.14.4101-4103.2000 PMC94598

[CIT0084] Yang C, Wan MT, Lauderdale TL, Yeh KS, Chen C, Hsiao YH, Chou CC. 2017. Molecular characteristics of clinical methicillin-resistant *Staphylococcus pseudintermedius* harboring arginine catabolic mobile element (ACME) from dogs and cats. Vet J. 224:46–49. 28697875 10.1016/j.tvjl.2017.06.002

[CIT0085] Yoon JW, Lee GJ, Lee SY, Park C, Yoo JH, Park HM. 2010. Prevalence of genes for enterotoxins, toxic shock syndrome toxin 1 and exfoliative toxin among clinical isolates of *Staphylococcus pseudintermedius* from canine origin. Vet Dermatol. 21(5):484–489. 20500497 10.1111/j.1365-3164.2009.00874.x

[CIT0086] Youn JH, Koo HC, Ahn KJ, Lim SK, Park YH. 2011. Determination of staphylococcal exotoxins, SCCmec types, and genetic relatedness of *Staphylococcus intermedius* group isolates from veterinary staff, companion animals, and hospital environments in Korea. J Vet Sci. 12(3):221–226. 21897094 10.4142/jvs.2011.12.3.221 PMC3165150

[CIT0087] Zecconi A, Scali F. 2013. *Staphylococcus aureus* virulence factors in evasion from innate immune defenses in human and animal diseases. Immunol Lett [Internet]. 150(1–2):12–22. 10.1016/j.imlet.2013.01.004 23376548

